# Analyzing the reduction quality of the distal radioulnar joint after closed K-wire transfixation in a cadaver model: is supination or neutral position superior?

**DOI:** 10.1007/s00402-023-05181-6

**Published:** 2024-03-05

**Authors:** Jan Siad El Barbari, Laura Kohlhas, Jochen Franke, Paul Alfred Grützner, Marc Schnetzke, Benedict James Swartman

**Affiliations:** 1 BG Klinik Ludwigshafen, Department for Orthopaedics and Trauma Surgery, Clinic at Heidelberg University, Ludwig-Guttmann-Str. 13, 67071 Ludwigshafen, Germany; 2 Institute of Medical Biometry, University of Heidelberg, Heidelberg, Germany; 3 German Joint Center Heidelberg, ATOS Clinic Heidelberg, Heidelberg, Germany

**Keywords:** Distal radioulnar joint instability, K-wire transfixation, Radioulnar ratio, Cadaveric study, Distal radius fracture

## Abstract

**Introduction:**

Distal radioulnar joint (DRUJ) instabilities are challenging and their optimal treatment is controversial. In special cases or when reconstruction of the stabilizing triangular fibrocartilage complex (TFCC) fails, K-wire transfixation can be performed. However, no consensus has been reached regarding the rotational position of the forearm in which this should be done. Therefore, it was investigated whether anatomical reduction would best be achieved by transfixation in neutral position or supination of the forearm.

**Materials and methods:**

Twelve cadaveric upper limbs were examined before dissection of the DRUJ stabilizing ligaments and after closed transfixation in both positions by C-arm cone-beam CT. Whether this was first done in neutral position or in supination was randomized. The change in the radioulnar ratio (RR) in percentage points (%points) was analyzed using Student's *t-*test. RR was used since it is a common and sensitive method to evaluate DRUJ reduction, expressing the ulnar head's position in the sigmoid notch as a length ratio.

**Results:**

The analysis showed an increased change in RR in neutral position with 5.4 ± 9.7%points compared to fixation in supination with 0.2 ± 16.1%points, yet this was not statistically significant (*p* = 0.404).

**Conclusions:**

Neither position leads to a superior reduction in general. However, the result was slightly closer to the anatomical position in supination. Thus, transfixation of the DRUJ should be performed in the position in which reduction could best be achieved and based on these data, that tends to be in supination. Further studies are necessary to validate these findings and to identify influential factors.

## Introduction

Distal radioulnar joint (DRUJ) instability is a common and challenging injury [1]. Although they occur in up to 10–40% of distal radius fractures (DRF) clinical diagnosis is still often missed, and chronic ulnar-sided pain and impaired function occur [2–6]. In Galeazzi fractures or Essex-Lopresti injuries (ELIs), this instability is always present, in these cases, as well as if ultimately diagnosed in DRF, the primary goal would be reconstruction of the stabilizing ligaments which form the triangular fibrocartilage complex (TFCC). Whether this should be done arthroscopically or open depends on the kind and extent of the damage. This can be done using suture anchors for ligamental damage or screw osteosynthesis in case of a fractured styloid process of the ulnar head [7–9]. If this is unsuccessful or not indicated, i.e. arthrosis of the DRUJ, K-wire transfixation might be performed [9]. However, no consensus or striking evidence exists on whether anatomical reduction is best achieved in supination or neutral position [10–13]. Some recommendations prefer transfixation in supination since most fractures are extension fractures with a dorsal subluxation that could best be reduced by this [10]. Others prefer reduction in neutral position because most work environments, i.e., computer work, need a full pronation; thus, impairment of this should primarily be reduced [14]. Immobilization or K-wire transfixation in pronation is commonly regarded as the worst option, leading to a significant and difficult-to-compensate loss of supination. It is therefore not discussed as a third option [15].

Due to the challenging clinical diagnosis, many methods have recently been described to objectify articulation in the DRUJ using CT scans and cone-beam CT (CBCT) [16–20]. Because of its feasibility and high intra- and interrater reliability, the most frequently used method is the radioulnar ratio (RR), as described by Lo et al. 2001. It assesses radioulnar translation as a ratio between the distance of the center of the ulnar head to the dorsal edge of the sigmoid notch and the length of the sigmoid notch itself (Fig. 2). Previous studies demonstrated the validity of this method as an intraoperative diagnostic tool [21, 22].

In most cases, the DRUJ is examined clinically for a remaining instability after the DRF has been cared for by volar plating in supination [13]. If subluxation of the ulna can be provoked, the DRUJ will be manually reduced and radioulnar transfixation with K-wires can be considered to hold the reduction and allow proper healing under immobilization [23, 24]. Yet controversy remains as to whether it should be done in supination or neutral position. [1, 7, 8, 10, 25–36].

In order to shed light on this controversial debate, CBCTs before and after performing closed transfixation of the DRUJ in supination and neutral position in 12 cadaveric forearms were taken and the anatomical reduction was analyzed and compared by comparing the change in the RR after transfixation between both positions.

The study hypothesis was that anatomical reduction should preferably be achieved in supination rather than in neutral position.

## Materials and methods

### Specimens

12 complete unpaired human upper limp specimens were examined without any macroscopic or radiologic defect. The specimens were provided by the anatomical department of the University of Graz, Austria. The local ethical board (Rhineland-Palatinate Local Ethics Committee, 55019 Mainz, Germany, No. 837.299.17) gave ethical approval. The sex of the specimens was unknown and their mean age was 74 years (range 68 to 84 years). Fixation of the limbs was performed using Thiel's procedure, which offers realistic conditions in regard to consistency, elasticity, and color [37, 38].

### Dissection and transfixation

After the initial 3D scan had been obtained an ELI was produced following the described technique by Kachooei [39] by a single operator (B.S.) as described in detail in a preliminary study [21]. Summarized, the palmar and dorsal distal radioulnar ligament was dissected and the TFCC was separated from its insertion at the ulnar styloid. Then the interosseous membrane (IOM) was split over its entire length and the radial head was resected under preservation of the distal biceps tendon.

**Fig. 1:**
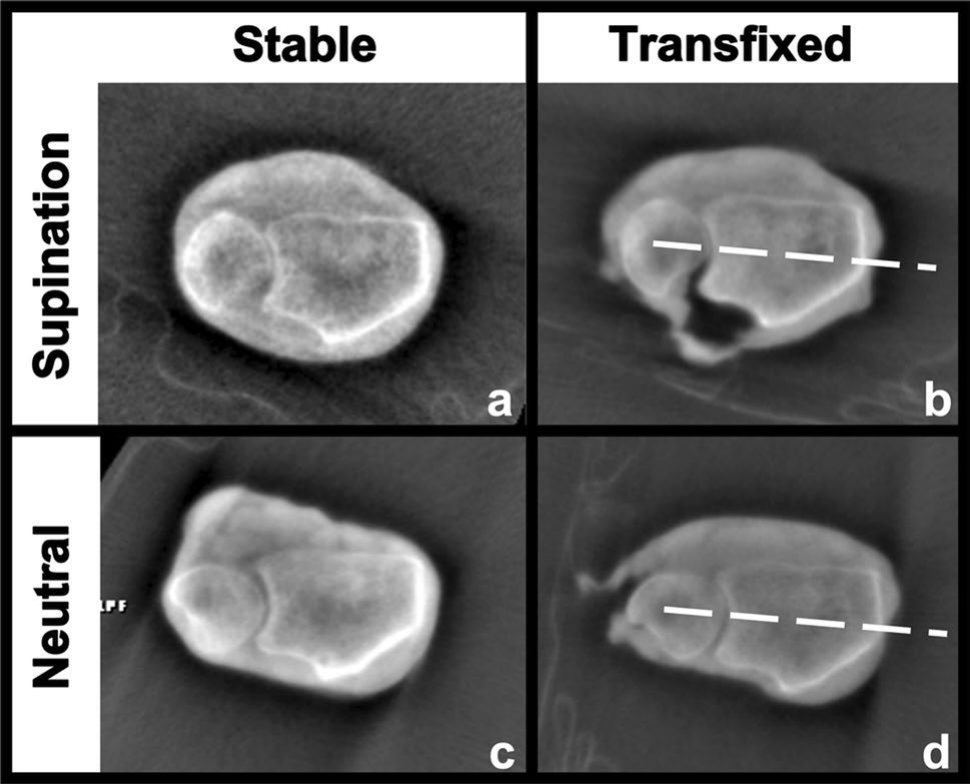
Axial planes of specimen 3 used for measurements with **a** positioned in supination before dissection, **b** after transfixation in supination, **c** positioned in neutral position, **d** after transfixation in neutral position. Dashed line symbolizing K-wire transfixation

Afterwards, closed transfixation of the DRUJ of the specimens was performed by B.S. using a single K-wire (2.0 mm) in both supination and neutral position (Fig. 1). The sequence in which this was done, either first in supination or in neutral position, was randomized.

### Imaging

**Fig. 2:**
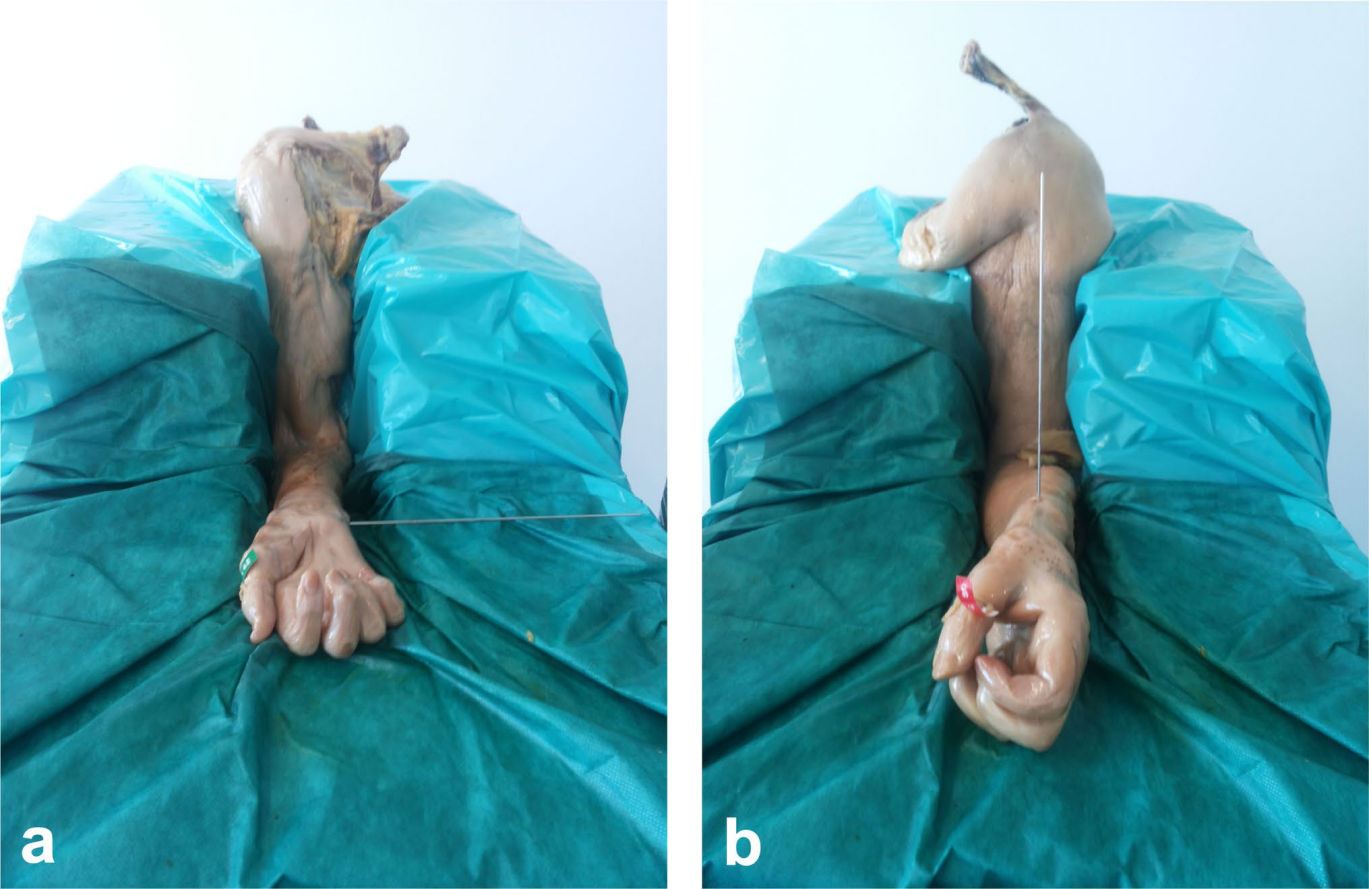
Example of the experimental setup after transfixation in **a** supination and **b** neutral position

A custom-made device was used to fixate the specimens in 90° flexion while allowing full pronosupination of the forearm (Fig. 2). Angles were measured by using a goniometer.

A CBCT of the wrist was taken in the uninjured specimens and after transfixation in either supination or neutral position using a 3D-capable C-Arm (Arcadis Orbic, Siemens Healthcare GmbH, Erlangen, Germany). These C-arms perform a motorized isocentric orbital rotation acquiring up to 400 images of the subject, calculating an editable 3D volume. In this volume, the planes can be freely manually adjusted and were reconstructed according to the suggested method by Park et al. and Wijffels et al. [19, 40]. The axial plane was adjusted 90° to the radial shaft, depicting the sigmoid notch's largest diameter in its center and showing both the Lister's tubercle and the ulnar styloid process.

### Measurement

**Fig. 3:**
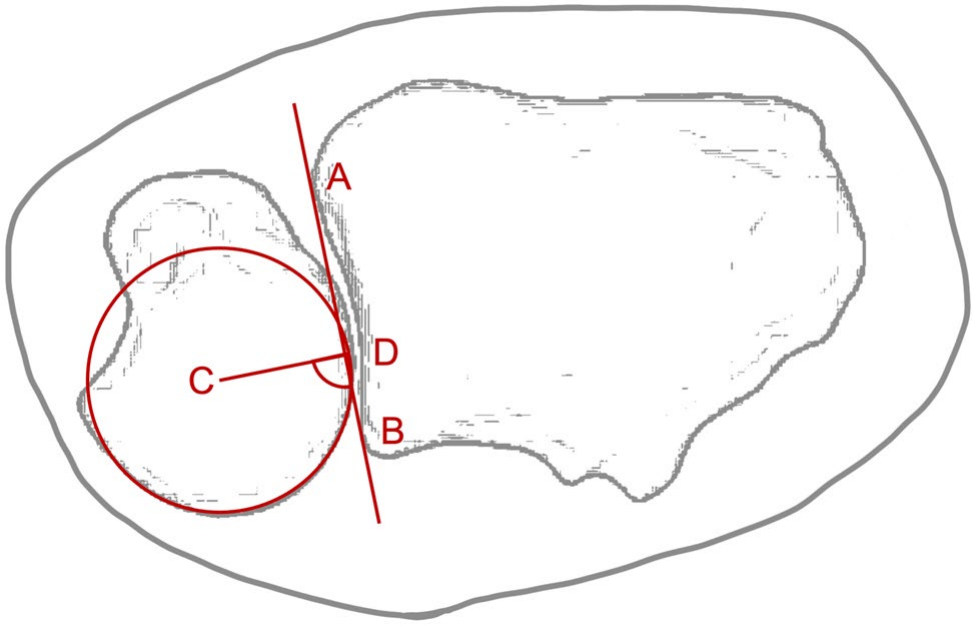
Measurement of the radioulnar ratio by the method of Lo et al. AB shows the sigmoid notch. CD marks the perpendicular line to AB through the center of the ulnar head. The length of AD is related to AB

As diligently described in preliminary studies, the RR method by Lo et al. was used, as depicted in Fig. 3 [18, 21, 22]. Briefly, this method quantifies radioulnar position by the ratio between the distance of the center of the ulnar head to the dorsal sigmoid notch and the length of the sigmoid notch. Measurements were performed for supination as well as neutral position before dissection and after transfixation on printed radiographs after magnifying them 1.5 times.

The difference between both values in percentage was calculated to quantify the difference in anatomical reduction using

1 $${\text{RR }}\left[ { {\text{\%points}}} \right]{\text{ }} = {\text{ RR}}_{{{\text{stable}}}} \left[ \% \right] - {\text{ RR}}_{{{\text{transfixed}}}} \left[ \% \right].$$

## Statistics

The mean change in the value of the RR (ΔRR; as calculated above) and its standard deviation were calculated in both supination and neutral position to analyze how closely anatomical reduction had been achieved. Paired *t*-tests were used to compare RR between stable and transfixed DRUJ, as well as ΔRR between neutral position and fixation in supination. Due to the study's exploratory nature, p-values are descriptive.

## Results

**Fig. 4:**
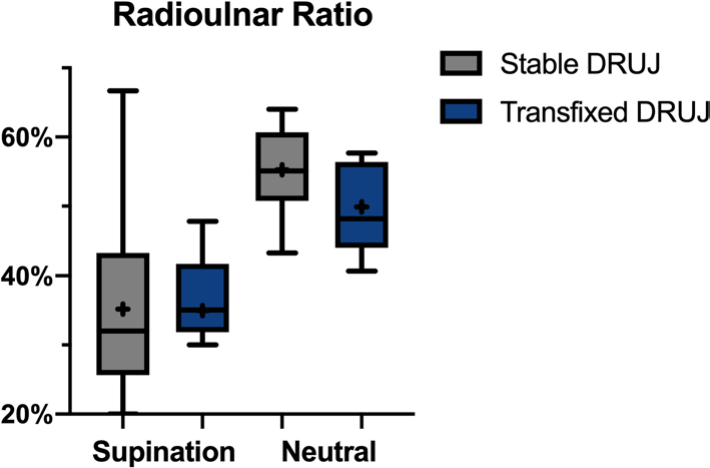
Comparison of the calculated Radioulnar Ratio in the stable and the transfixed DRUJ

**Fig. 5:**
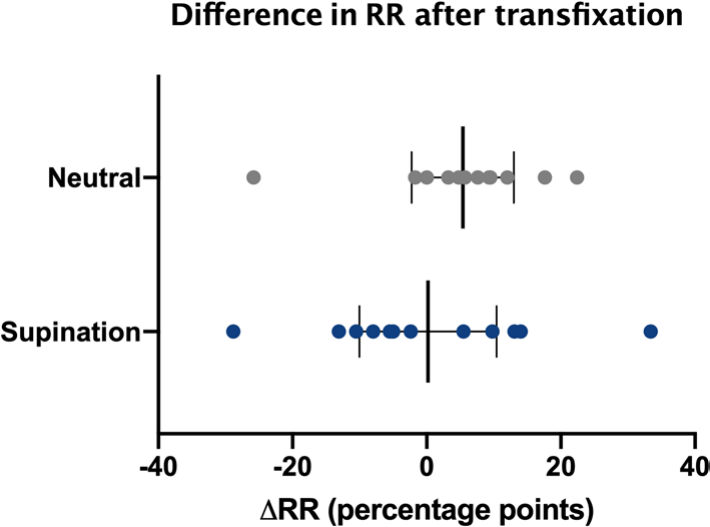
Difference in the calculated RR before and after transfixation in neutral and supinated position

The descriptive statistics and analyses with the mean values and standard deviations are shown in Table 1. Statistical analysis showed no significant difference in RR for either transfixation in supination or in neutral position (*p*_supination_ = 0.965; *p*_neutral_ = 0.148; Fig. 4). In supination, the mean ΔRR was 0.2 percentage points (SD 16.1; 95% CI [− 10.0; 10.4]) with 35.2% (SD 13.0; 95% CI [27.0;43.4]) before dissection and 35.0% (SD 9.7; 95% CI [28.8; 41.1]) after transfixation. The mean difference in neutral position was somewhat higher with 5.4%points (SD 12.0; 95% CI [− 2.2; 13.0]) with 55.3% (SD 6.5; 95% CI [51.2; 59.4]) vs. 49.9% (SD 13.0; 95% CI [41.6; 58.2]), respectively. But in statistical analysis, the ΔRR was not significantly different between both groups (*p*_Δ_ = 0.404; Fig. 5).

**Table 1:** Resulting mean (standard deviation) of Radioulnar Ratio (RR) values and the difference in percentage points in both supination and neutral position with p-values calculated by Student’s *t-test*. 95% confidence intervals are given for each value

	RR_stable_ [%]	RR_transfixed_ [%]	*p*-value
Supination 95% CI	35.2 (13.0) [27.0; 43.4]	35.0 (9.7) [28.8; 41.1]	0.965
Neutral 95% CI	55.3 (6.5) [51.2; 59.4]	49.9 (13.0) [41.6; 58.2]	0.148

	Supination	Neutral	*p*-value
ΔRR [%points] 95% CI	0.2 (16.1) [−10.0; 10.4]	5.4 (12.0) [−2.2; 13.0]	0.404

## Discussion

It could be shown that in the case of closed transfixation in an unstable DRUJ, supination tends to offer a better anatomical reduction as assessed by the RR method using CBCT. Performing closed reduction after producing an ELI instead of a DRF was chosen, because it was hypothesized that if there was a difference it should become apparent in the most unstable injury pattern at least. Reducing the risk of a false-negative result.

In a previous study, it could be demonstrated that CBCT is a reliable and valid method to assess radioulnar migration by using the RR method in uninjured and dissected cadaver specimens [21, 22]. Methods to objectively assess the DRUJ were developed to lower the rate of missed diagnoses since the clinical assessment is unreliable and often leads to chronic instability and pain with substantial loss of function [2, 3, 20, 40]. Besides the RR method, several other methods are described, namely, Radioulnar Line, the Subluxation Ratio, and the Epicenter method [40]. We used the RR method because it has the best feasibility, highest sensitivity and specificity, and, as it is the most consistently used in recent literature, the best comparability [22].

Treatment of instabilities of the DRUJ is very heterogenic. Historically they have often been treated by sole immobilization with unsatisfactory outcomes. Then methods using K-wire transfixation had been developed but current gold standard would be reconstruction of the TFCC as good as possible, especially in patients with a high functional demand [9]. However, if reconstruction fails or if, in special cases, it is not indicated K-wire transfixation might still be performed. Mikic performed temporary radioulnar K-wire transfixation after osteosynthesis of the radial fracture if DRUJ instability remained and saw excellent outcomes in all study patients [15, 24, 41]. However, he did not describe in which position transfixation had been performed. Since then, many studies have been performed, all confirming a better outcome after transfixation. However, it was often not stated in which position transfixation had been performed or whether positioning changed between supination and neutral position [42, 43].

The primary goal in immobilizing joints is to choose a position in which the ligaments and tendons are maximally taut to avoid resulting contractions and a diminished range of motion. Hence, several anatomical studies were conducted in the last three decades to evaluate in which position this would be the case for the stabilizing ligaments and tendons of the forearm, namely, the TFCC with the palmar and dorsal radioulnar ligaments and the IOM [25–36]. The current consensus would be adequately described by stating that in regard to the TFCC in supination, the superficial palmar and the deep dorsal parts are under tension and vice versa in pronation. The latest study on the IOM from Razak et al. claims that there are indeed differences in relative numbers in tension of the IOM in different rotational positions, yet in absolute numbers, the differences would be negligible. They claim it primarily functions as a stabilizer against longitudinal forces [36].

Until now, many studies have been performed to analyze the outcome after transfixation in either supination or neutral position. Yet, to our knowledge, no study has ever performed transfixation in both positions to compare their potential for anatomical reduction.

Fixation in supination had been performed by Rettig et al. with an excellent result in 95% of 40 patients [44]. In this study, DRUJ instabilities were treated with an external fixator in supination, leading to an improved range of motion regarding supination and no higher rate of disability [45]. Schnetzke et al. also performed transfixation in supination in ELIs and compared these early-diagnosed and properly treated ELIs with late-diagnosed ones [2]. They saw a significant improved outcome in the group that received transfixation in supinated position. In their study, Giannoulis et al. report that in unstable DRUJs transfixation is mostly preferred in neutral position to minimize a possible loss of pronation [15]. Their suggested method in children is choosing the position depending on the direction in which the joint dislocates. In dorsal subluxation, the joint should be reduced and fixated in supination, and in volar subluxation, it should be done in neutral position. Fixation in pronation should be avoided because of the danger of causing a loss of supination with significant impaired function, which is difficult to compensate [15, 46]. In adults, however, they advise transfixation in neutral or only slight supination. A similar suggestion is made by Azimi and Wysocki in their review, stating that the exact position of the forearm is not as important as adequate reduction of the ulnar head within the sigmoid notch [23].

Fixation in supination seems to be slightly superior according to the following factors. Since radial fractures often occur as extension fractures leading to a dorsal subluxation of the ulna, Kihara et al. stated that because this occurs in supination as well as the best reduction can be achieved with the forearm supinated, the DRUJ should be transfixed in this position [26]. Similarly, Jung et al. found a favorable outcome after immobilization in semisupination in case of a dorsal instability compared to neutral position [10]. Sammer et al. came to a similar conclusion, saying that the DRUJ should be stabilized in the position in which it is firmest, which is most commonly supination [12]. This is additionally supported by the data of Gupta et al. [32]. They measured the radioulnar migration after dissection of the ligamentous stabilizers and found a significantly increased translation in pronation but not in supination.

Our results show no significant difference between the RR before and after transfixation in either supination or neutral position, as shown in Fig. 4. This indicates that the remaining controversy exists because neither version is clearly superior. However, as Fig. 5 demonstrates, reduction and fixation in supination achieved a more anatomical value than fixation in neutral position in which a relevantly increased difference in ΔRR in comparison to fixation in supination became apparent.

Thus, transfixation should be performed primarily in the position in which reduction can be best or most firmly achieved, but when in doubt, supination might be preferred for the following two reasons: First, DRFs are usually extension fractures and dorsal subluxation is more likely to be treated better in supination, and second, although these findings were not significant, the reduction achieved tended to be more anatomic in supination.

### Limitations

The main limitation of this study is that a biomechanic aspect in cadaveric specimens was examined; therefore, the extent to which dynamic soft tissue stabilizers would influence the findings cannot be specified. Yet, as previous studies have shown, Thiel's method is supposed to embalm the soft tissues so that these retain similar properties to those shown in clinical settings. Therefore, it was considered a reliable method for use in this study. These findings are also limited by the mean age of 74 years in our study population that does not resemble the general population. Nonetheless, we successively compared the RR before and after dissection and transfixation, so the extent to which they differ should not be influenced by age. The small study group of only 12 specimens also affects the validity of this study. However, prior cadaveric studies have shown a similar sample size and are generally accepted as sufficient [19, 22, 40].

## Conclusion

Summarizing all previous studies and adding these findings our conclusion regarding DRUJ transfixation is as follows:

- DRUJ transfixation in neutral position as well as in supination can lead to comparable results regarding anatomical DRUJ alignment.
- Choosing the proper positioning of the DRUJ for transfixation, specific workplace requirements, as well as DRUJ reduction must be considered.

Thus, if a primary reconstruction of the TFCC was unsuccessful and K-wire transfixation would be performed, supination tends to offer a better anatomical reduction and should therefore be recommended, if in doubt, in accordance with these results in closed transfixation. Further studies are necessary to validate our recommendation and to identify further influential factors.

## Data Availability

The images and datasets used and analysed during the current study are available from the corresponding author on request.

## References

[1] Giddins G (2023). The distal radioulnar joint after distal radial fractures: when and how do we need to treat pain, stiffness or instability?. J Hand Surg.

[2] Schnetzke M (2017). Outcome of early and late diagnosed essex-lopresti injury. J Bone Joint Surg Am.

[3] Lindau T (2000). Distal radioulnar instability is an independent worsening factor in distal radial fractures. Clin Orthopaed Related Res.

[4] May MM, Lawton JN, Blazar PE (2002). Ulnar styloid fractures associated with distal radius fractures: incidence and implications for distal radioulnar joint instability. J Hand Surg.

[5] Nellans KW, Kowalski E, Chung KC (2012). The epidemiology of distal radius fractures. Hand Clin.

[6] Pickering GT (2022). The reliability of clinical assessment of distal radioulnar joint instability. J Hand Surg Eur.

[7] Mau M (2022). Optimizing the orientation of a suture button to stabilize the distal radioulnar joint in a sawbones model. J Hand Surg Global Online.

[8] Xiao AX (2021). Management of acute distal radioulnar joint instability following a distal radius fracture: a systematic review and meta-analysis. J Hand Surge Global Online.

[9] Spies CK (2021). Die Therapie des instabilen distalen Radioulnargelenkes (DRUG). Handchirurgie Scan.

[10] Jung H-S (2022). Postoperative immobilization using a short-arm cast in the semisupination position is appropriate after arthroscopic triangular fibrocartilage complex foveal repair. Bone Joint J.

[11] Rhee PC, Shin AY (2016). Management of complex distal radius fractures: review of treatment principles and select surgical techniques. J Hand Surg Asian Pac.

[12] Sammer DM, Chung KC (2012). Management of the distal radioulnar joint and ulnar styloid fracture. Hand Clin.

[13] Spies CK (2020). Distal radioulnar joint instability: current concepts of treatment. Arch Orthop Trauma Surg.

[14] Park MJ (2012). Immobilization in supination versus neutral following surgical treatment of Galeazzi fracture-dislocations in adults: case series. J Hand Surg Am.

[15] Giannoulis FS, Sotereanos DG (2007). Galeazzi fractures and dislocations. Hand Clin.

[16] Iida A (2012). Distal radioulnar joint stress radiography for detecting radioulnar ligament injury. J Hand Surg Am.

[17] Kim JP, Park MJ (2008). Assessment of distal radioulnar joint instability after distal radius fracture: comparison of computed tomography and clinical examination results. J Hand Surg Am.

[18] Lo IK (2001). The radioulnar ratio: a new method of quantifying distal radioulnar joint subluxation. J Hand Surg Am.

[19] Park MJ, Kim JP (2008). Reliability and normal values of various computed tomography methods for quantifying distal radioulnar joint translation. J Bone Joint Surg Am.

[20] Scheer JH, Hammerby S, Adolfsson LE (2010). Radioulnar ratio in detection of distal radioulnar joint instability associated with acute distal radius fractures. J Hand Surg Eur.

[21] Swartman B (2019). Distal radioulnar joint instability with three different injury patterns assessed by three-dimensional C-arm scans: a cadaveric study. J Hand Surg Eur.

[22] Swartman B (2019). Normal values of distal radioulnar translation assessed by three-dimensional C-arm scans: a cadaveric study. J Hand Surg Eur.

[23] Azimi HJ, Wysocki RW (2022). Distal radioulnar joint instability and Galeazzi fractures. Skeletal trauma of the upper extremity.

[24] Mikic ZD (1975). Galeazzi fracture-dislocations. J Bone Joint Surg Am.

[25] Rabinowitz RS (1994). The role of the interosseous membrane and triangular fibrocartilage complex in forearm stability. J Hand Surg Am.

[26] Kihara H (1995). The stabilizing mechanism of the distal radioulnar joint during pronation and supination. J Hand Surg Am.

[27] Skahen JR (1997). The interosseous membrane of the forearm: anatomy and function. J Hand Surg Am.

[28] Nakamura T, Yabe Y, Horiuchi Y (1999). Functional anatomy of the interosseous membrane of the forearm—dynamic changes during rotation. Hand Surg.

[29] Nakamura T, Yabe Y, Horiuchi Y (1999). In vivo MR studies of dynamic changes in the interosseous membrane of the forearm during rotation. J Hand Surg Br.

[30] Manson TT (2000). Forearm rotation alters interosseous ligament strain distribution. J Hand Surg Am.

[31] Pfaeffle HJ (2000). Role of the forearm interosseous ligament: is it more than just longitudinal load transfer?. J Hand Surg Am.

[32] Gupta R (2002). Kinematic analysis of the distal radioulnar joint after a simulated progressive ulnar-sided wrist injury. J Hand Surg Am.

[33] Farr LD (2015). Anatomy and biomechanics of the forearm interosseous membrane. J Hand Surg Am.

[34] Werner FW (2017). Role of the interosseous membrane in preventing distal radioulnar gapping. J Wrist Surg.

[35] Hojo J (2019). Three-dimensional kinematic analysis of the distal radioulnar joint in the axial-loaded extended wrist position. J Hand Surg Am..

[36] Bin Abd Razak HR (2020). An anatomical and biomechanical assessment of the interosseous membrane of the cadaveric forearm. J Hand Surg Eur.

[37] Thiel W (1992). The preservation of the whole corpse with natural color. Ann Anat.

[38] Vollner F (2017). Stability of knee ligament complex of Thiel-embalmed cadaver compared to in vivo knee. J Mech Behav Biomed Mater.

[39] Kachooei AR (2015). Intraoperative physical examination for diagnosis of interosseous ligament rupture-cadaveric study. J Hand Surg Am.

[40] Wijffels M (2016). Computed tomography for the detection of distal radioulnar joint instability: normal variation and reliability of four CT scoring systems in 46 patients. Skeletal Radiol.

[41] Geissler WB, Fernandez DL, Lamey DM (1996). Distal radioulnar joint injuries associated with fractures of the distal radius. Clin Orthopaed Related Res.

[42] Lindau T, Aspenberg P (2002). The radioulnar joint in distal radial fractures. Acta Orthop Scand.

[43] Mader K, Pennig D (2006). The treatment of severely comminuted intra-articular fractures of the distal radius. Strat Trauma Limb Reconstr.

[44] Rettig ME, Raskin KB (2001). Galeazzi fracture-dislocation: a new treatment-oriented classification. J Hand Surg Am.

[45] Ruch DS, Lumsden BC, Papadonikolakis A (2005). Distal radius fractures: a comparison of tension band wiring versus ulnar outrigger external fixation for the management of distal radioulnar instability. J Hand Surg Am.

[46] Rodriguez-Merchan EC, Shojaie B, Kachooei AR (2022). Distal radioulnar joint instability: diagnosis and treatment. Arch Bone Jt Surg.

